# A case report of bevacizumab-induced subcapsular renal hemorrhage in a patient with lung adenocarcinoma

**DOI:** 10.3389/fonc.2026.1804964

**Published:** 2026-05-19

**Authors:** Yue Du, Han Yu, Fei-Yan Zhou, Di Xu, Fang-Hua Song

**Affiliations:** 1 Xinhua Hospital, Dalian University, Liaoning, Dalian, China; 2 2nd Tumor Ward, Xinhua Hospital, Dalian University, Liaoning, Dalian, China

**Keywords:** adverse drug reaction, bevacizumab, lung adenocarcinoma, oncology, subcapsular renal hemorrhage

## Abstract

This article reports a case of bilateral renal subcapsular hemorrhage in a 51-year-old female patient with lung adenocarcinoma and multiple metastases after receiving four cycles of combination antitumor therapy with pemetrexed, carboplatin, sintilimab, and bevacizumab. The patient had a pre-existing cystic lesion in the right kidney and developed intermittent fever, hypertension, and anemia during treatment. The diagnosis was confirmed by a renal contrast-enhanced CT scan. Management involved immediate discontinuation of bevacizumab, absolute bed rest, hemostasis, antihypertensive therapy, and other comprehensive treatments. The renal hemorrhage was effectively controlled and gradually resolved. Based on the temporal relationship of medication use and clinical manifestations, the renal hemorrhage was considered to be associated with bevacizumab. In recent years, bevacizumab has been widely used in tumor treatment; however, its adverse effects still require further exploration. Hemorrhage induced by bevacizumab primarily occurs in the respiratory tract, digestive tract, and brain, while renal subcapsular hemorrhage has not been reported. This case suggests that bleeding manifestations caused by bevacizumab are diverse and remain a cause for vigilance.

## 1 Introduction

Bevacizumab, as a humanized anti-vascular endothelial growth factor (VEGF) monoclonal antibody, exerts its therapeutic effect by specifically binding to VEGF and inhibiting tumor angiogenesis. It has become an important treatment modality for various solid tumors, including colorectal cancer and non-small cell lung cancer (NSCLC) (1). In lung adenocarcinoma, a major subtype of NSCLC, current treatment has evolved into a multidisciplinary paradigm. Depending on disease stage and molecular subtype, options include curative surgery, chemoradiotherapy, EGFR−TKI−targeted therapy, and immune checkpoint inhibitors primarily targeting PD−1/PD−L1. Appropriate use of these regimens has significantly improved patient prognosis (2–4). However, the clinical application of bevacizumab is accompanied by a series of vascular-related adverse effects, including hypertension, proteinuria, thromboembolic events, and bleeding tendencies (5). Among these, bleeding events are predominantly reported in mucosal and gastrointestinal hemorrhages, often closely associated with factors such as hypertension and tumor invasion of blood vessels (6). Subcapsular renal hemorrhage, occurring in a specific anatomical location, can clinically manifest as abdominal pain and a decline in hemoglobin levels, and is commonly associated with tumors, vascular diseases, and anticoagulant therapy (7). Although the drug labeling for bevacizumab explicitly warns of its bleeding risk, and some literature has reported associated epistaxis, gastrointestinal hemorrhage, and even intracranial bleeding, bevacizumab-induced and imaging-confirmed subcapsular renal hemorrhage has not been previously reported. This adverse event is clinically insidious, prone to misdiagnosis, delayed diagnosis or intervention, and may culminate in renal impairment or hemorrhagic shock in severe cases. Its precise pathophysiological mechanism remains incompletely elucidated. This article reports a case of bilateral subcapsular renal hemorrhage in a patient with lung adenocarcinoma treated with a bevacizumab-containing combination regimen, occurring in the absence of anticoagulant use, relevant trauma history, or other clear precipitating factors for bleeding. By systematically reviewing the patient’s clinical course and imaging data, this report analyzes the potential causes of bevacizumab-induced subcapsular renal hemorrhage, aiming to provide a reference for the early identification and clinical management of such severe adverse events.

## 2 Case presentation

**Figure 1:**
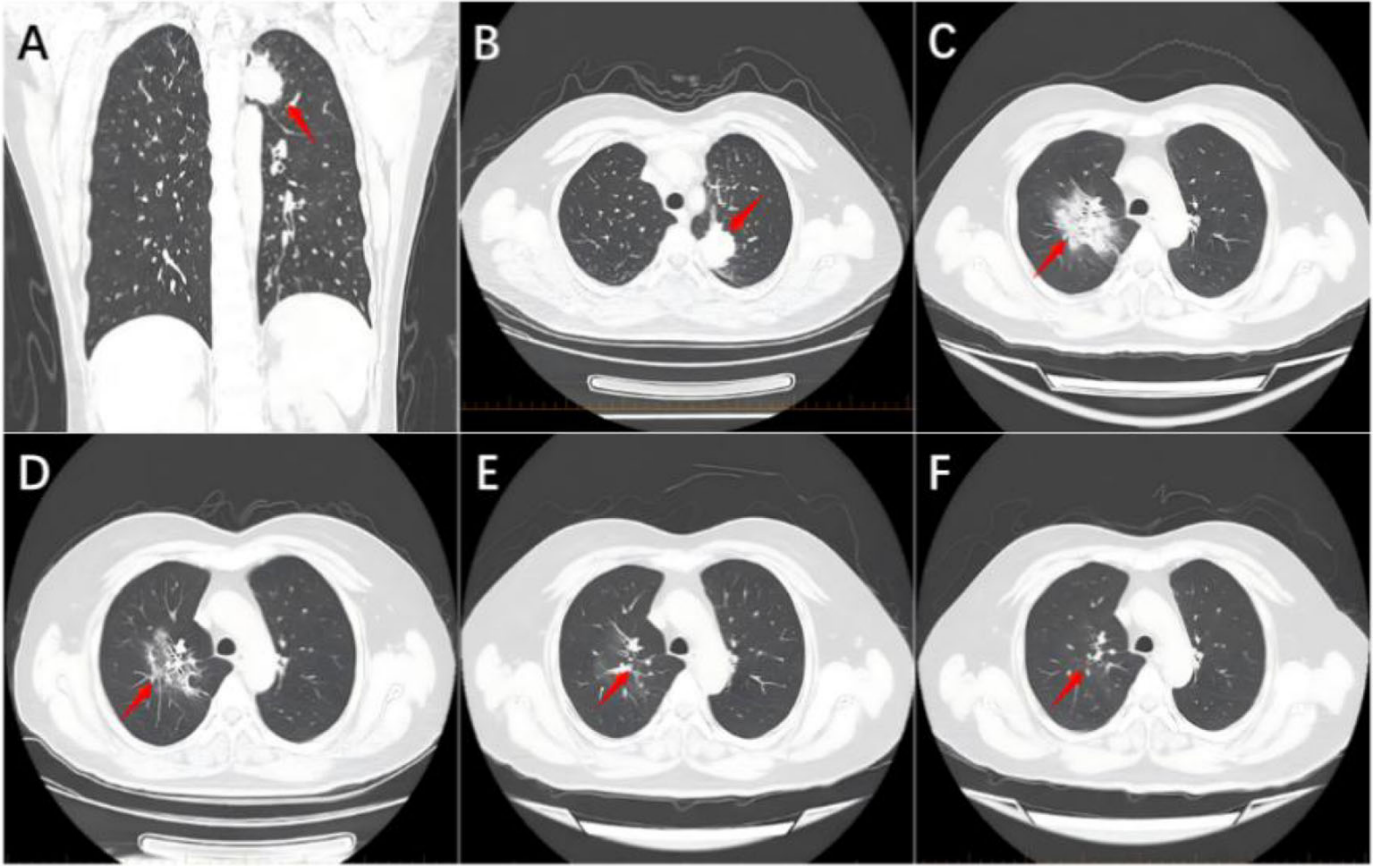
Series of chest CT images. **(A)** Coronal image from March 3, 2022, showing a mass approximately 3.1 cm × 2.9 cm in the left upper lobe (red arrow). **(B)** Axial image from March 3, 2022, showing the same mass in the left upper lobe (red arrow). **(C)** Image from April 14, 2025, showing a newly developed patchy high-density opacity approximately 5.4 cm × 3.7 cm in the right lung (red arrow). **(D)** Image from May 15, 2025, showing a persistent patchy focus in the right lung (red arrow). **(E)** Image from July 7, 2025, showing a patchy shadow in the right lung (red arrow). **(F)** Image from October 9, 2025, showing a patchy shadow in the right lung (red arrow).

**Figure 2:**
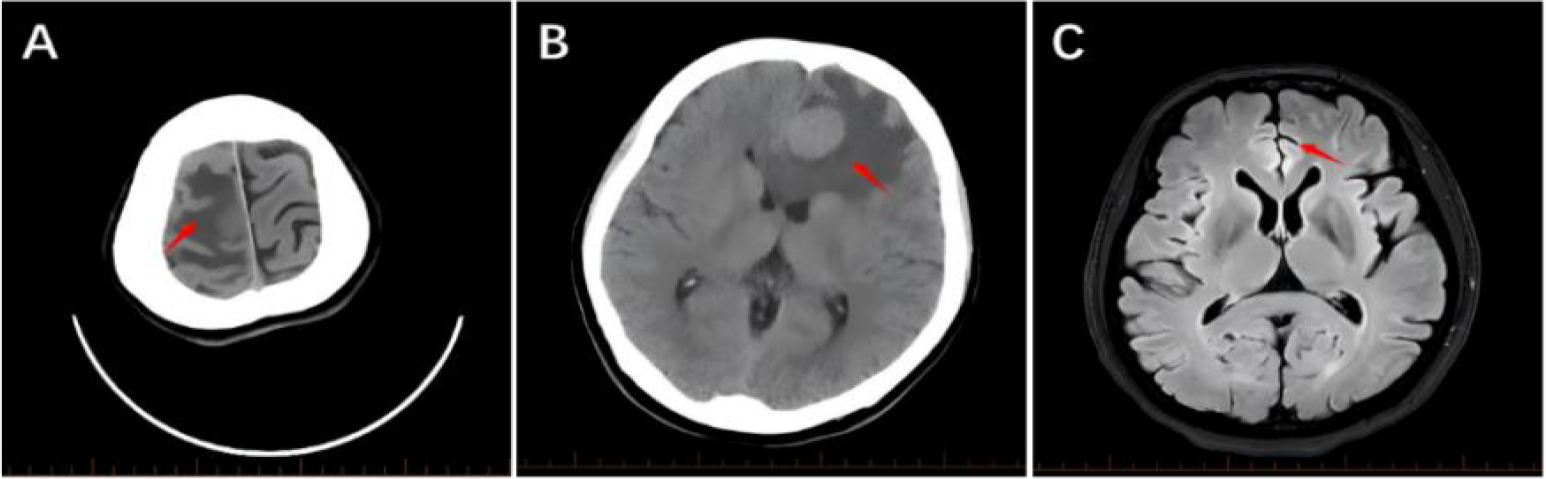
Neuroimaging. **(A)** Non-contrast head CT obtained on March 3, 2022, shows a hypodense lesion in the right cerebellar hemisphere (red arrow). **(B)** Follow-up non-contrast head CT on April 7, 2025, reveals a space-occupying lesion in the left frontal lobe (red arrow), measuring approximately 2.7 cm × 2.0 cm with an attenuation value of approximately 33 Hounsfield Units (HU), accompanied by perilesional edema. **(C)** Brain MRI performed on October 29, 2025, shows significant improvement compared to the study from April 7, 2025 (red arrow).

**Figure 3:**
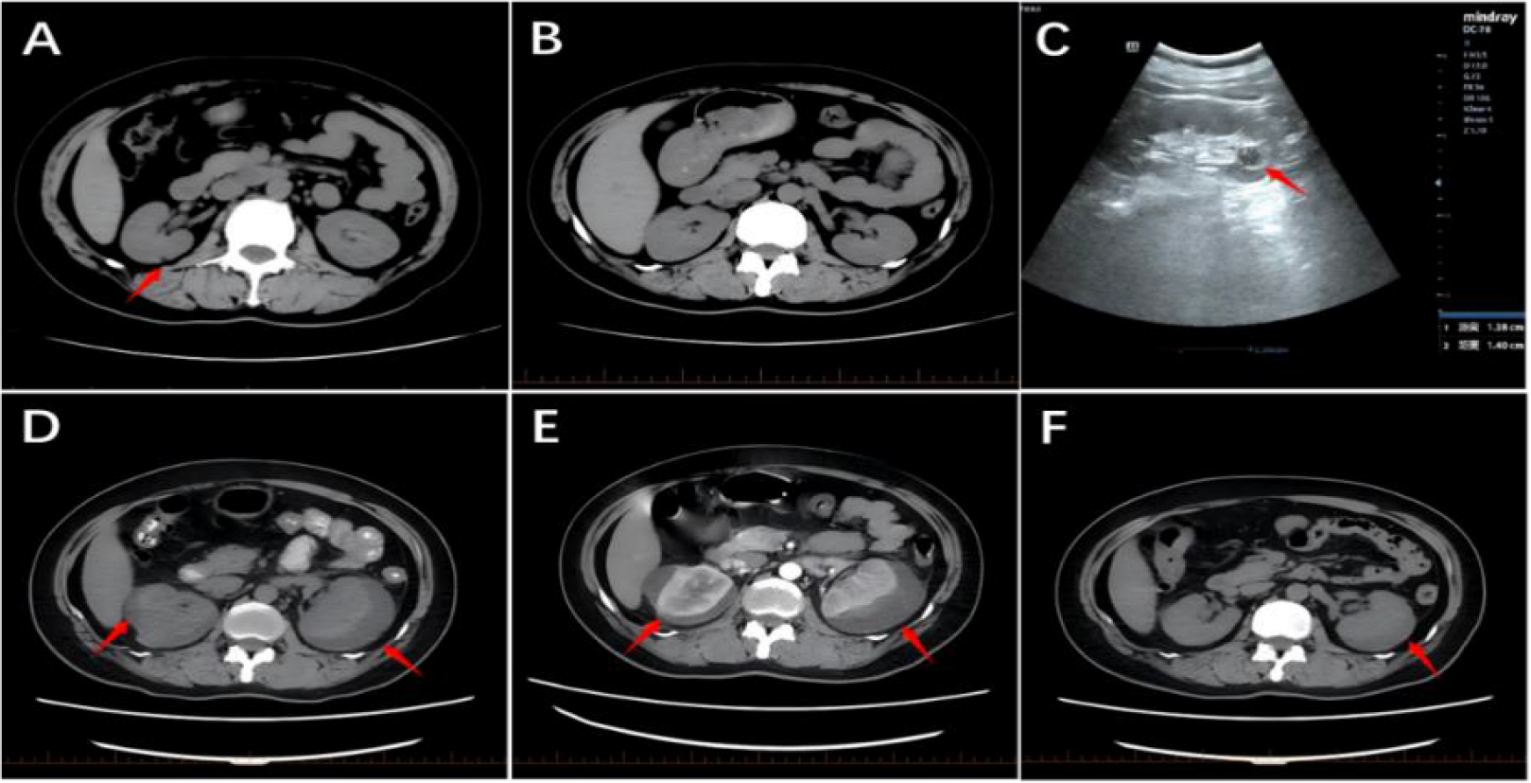
Abdominal imaging. **(A)** CT on March 3, 2022, shows a small cyst (approximately 0.4 cm, attenuation 2 HU) in the right kidney (red arrow). **(B)** CT on April 7, 2025, demonstrates no abnormalities in both kidneys. **(C)** Abdominal ultrasound on May 21, 2025, reveals a left renal cyst measuring approximately 1.38 cm × 1.40 cm (red arrow). **(D)** CT on July 7, 2025, indicates bilateral subcapsular hemorrhage and fluid collection, suggestive of hematoma (red arrow). **(E)** Contrast-enhanced CT on July 11, 2025, confirms bilateral subcapsular hemorrhage and fluid collection (red arrow). **(F)** CT on October 9, 2025, shows residual fluid collection in the left kidney (red arrow).

**Figure 4:**
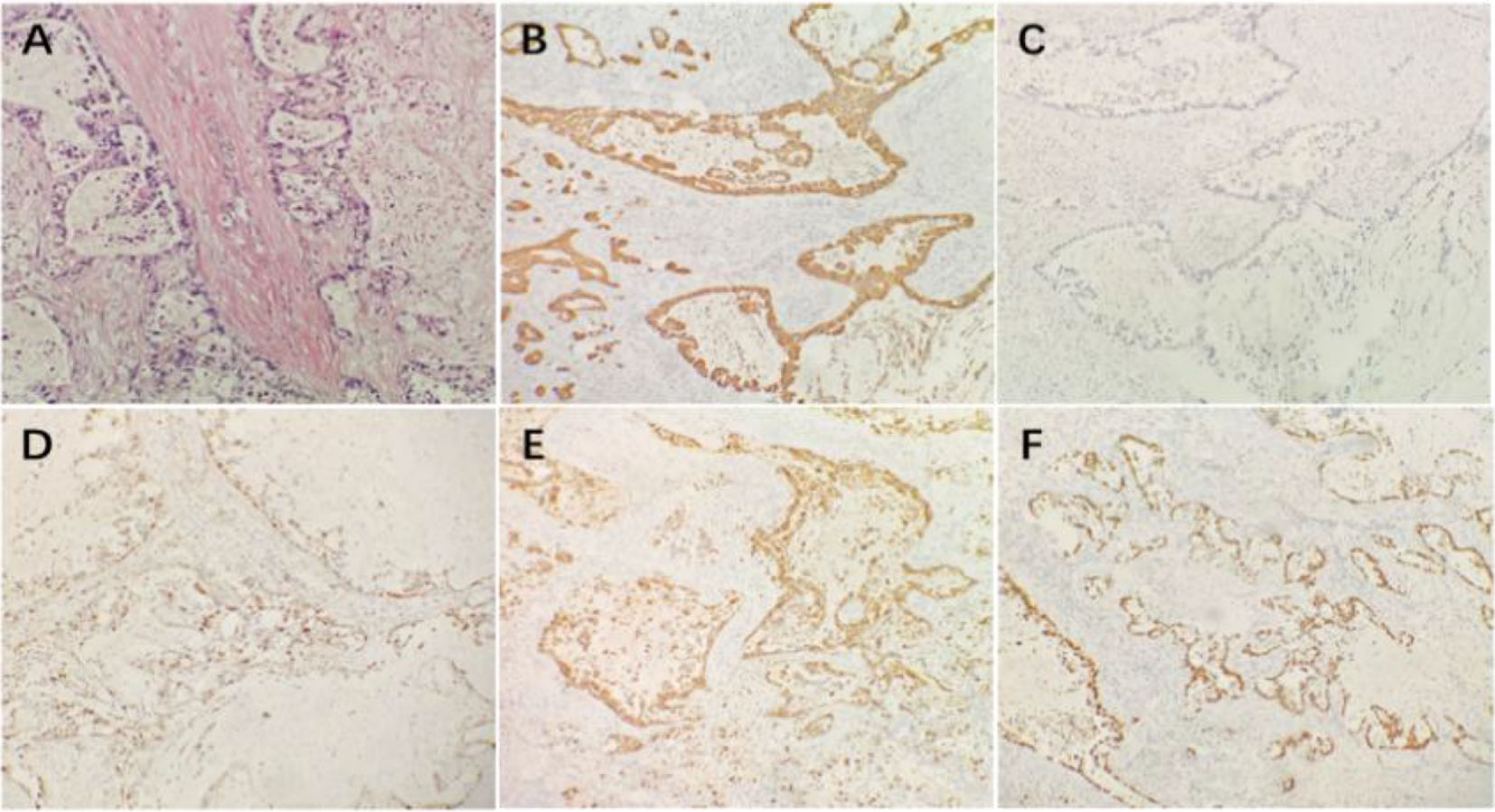
Pathological and immunohistochemical features of the cervical lymph node biopsy. **(A)** Hematoxylin−eosin **(HE)** staining shows infiltration of adenocarcinoma within the cervical lymph node tissue (×200). **(B)** CK7 immunostaining shows strong positivity (3+) in the tumor cells (×100). **(C)** CK20 immunostaining is negative in the tumor cells (×100). **(D)** Ki−67 immunostaining shows positivity in the tumor cells, with a proliferation index of approximately 20% (×100). **(E)** NapsinA immunostaining shows strong positivity (3+) in the tumor cells (×100). **(F)** TTF−1 immunostaining shows strong positivity (3+) in the tumor cells (×100).

A 51-year-old female patient presented in March 2022 due to progressively enlarged and tender left cervical lymph nodes discovered during a physical examination. CT scans revealed a space-occupying lesion in the left upper lobe of the lung (approximately 3.1cm × 2.9cm) (**Figures 1A, B**), a low-density focus in the right cerebellar hemisphere (**Figure 2A**), and a low-density cystic lesion within the right renal parenchyma (diameter 0.4cm, CT value approximately 2 HU) (**Figure 3A**). On March 4, 2022, a cervical lymph node biopsy confirmed metastatic lung adenocarcinoma (**Figure 4A**). Immunohistochemistry results were: CK7(3+); CK20(-); Ki-67(+20%); NapsinA (3+); TTF-1(3+) (**Figures 4B–F**). Genetic testing on the lymph node biopsy tissue indicated an EGFR exon 19 mutation. The patient underwent left lung surgery in May of the same year and subsequently received oral Furmonertinib targeted therapy. Follow-up evaluation in April 2025 revealed progression of bilateral pulmonary lesions and nodules, with the new appearance of a patchy opacity measuring approximately 5.4 cm × 3.7 cm in the right upper lobe (**Figure 1C**) and the development of brain metastasis (**Figure 2B**), no significant change is noted in the kidneys (**Figure 3B**). Due to tumor progression, the patient’s oral Furmonertinib was discontinued, and combination therapy with Pemetrexed 800mg d1 + Carboplatin 400mg d1 + Sintilimab 200mg d1 + Bevacizumab 400mg d1 was initiated on April 10, 2025, for four cycles (21 days per cycle). During treatment, follow-up chest CT on May 15, 2025(**Figure 1D**), compared with the prior scan from April 14, 2025, showed mild resolution with decreased density, suggestive of inflammatory changes. A routine ultrasound performed on May 21, 2025, revealed a left renal cyst measuring approximately 1.38 cm × 1.40 cm (**Figure 3C**). After completing four cycles of combination therapy, a chest CT on July 7, 2025, (**Figure 1E**) showed stable disease (SD) with shrinkage of lung lesions compared to May 15, 2025,but the patient developed intermittent fever (peak temperature 38°C), hypertension (blood pressure repeatedly >140/90 mmHg), and progressive hemoglobin decline (from 97 g/L to 93 g/L). An abdominal and pelvic CT (**Figure 3D**) suggested possible bilateral subcapsular renal fluid collections/hematomas. Laboratory tests indicated normal coagulation function and platelet count. Considering the patient’s recent Bevacizumab treatment history and after excluding other common causes of hemorrhage, such as trauma, nephritis, and renal calculi, a preliminary clinical diagnosis of “Bevacizumab-associated renal subcapsular hemorrhage” was made, based on new-onset hypertension during treatment, unexplained fever, progressive anemia, CT findings of bilateral subcapsular hyperdense hematomas, and exclusion of common causes including tumor rupture and coagulopathy. Given the multidisciplinary nature of the complication, a multidisciplinary team (MDT) involving medical oncology, urology, and radiology was convened to develop a comprehensive treatment plan. The MDT reached a consensus based on the drug label and NCI-CTCAE criteria: a Grade 3 bleeding event requires permanent discontinuation of bevacizumab. Therefore, bevacizumab was permanently stopped and conservative management was initiated. After bleeding stabilization, the following criteria must be met simultaneously before resuming chemotherapy and immunotherapy: hemodynamic stability (no active bleeding, blood pressure <140/90 mmHg), hemoglobin ≥90 g/L and stable for two weeks, imaging confirmation of stable hematoma without enlargement, and absence of signs of infection. Bevacizumab was not to be used in subsequent treatment. Specific therapeutic measures included strict bed rest to reduce perirenal tension and prevent further hemorrhage, hemostatic therapy with Carbazochrome Sodium Sulfonate combined with Tranexamic Acid, prophylactic antibiotics (Moxifloxacin) to prevent secondary infection of the hematoma or perirenal abscess formation, and blood pressure control with Amlodipine Besylate, along with supportive care and arrangements for further imaging studies. On July 11, 2025, a renal contrast-enhanced CT (**Figure 3E**) confirmed “bilateral subcapsular renal fluid collections/hematomas.” Given the association with Bevacizumab, this agent was not resumed in subsequent treatment. The patient continued with strict bed rest, closely monitoring blood pressure and urine output. As the renal hemorrhage was not fully resolved, the current cycle of anti-tumor therapy was suspended after discussion with the family, while oral antibiotic therapy was continued. Under close monitoring, the patient’s vital signs remained stable, hemoglobin levels stabilized at 102 g/L without further decline, and blood pressure (132/84 mmHg) was well-controlled, with no signs of active bleeding. After a comprehensive MDT assessment confirmed she met the criteria for restarting systemic anti-tumor therapy, the decision was made to initiate the fifth cycle of Pemetrexed + Carboplatin chemotherapy on August 14, 2025. On September 11, 2025, the patient received the sixth and seventh cycles of Pemetrexed chemotherapy combined with Sintilimab immunotherapy, with stable disease. After discontinuing Bevacizumab and following three months of conservative management, a follow-up CT showed improvement in the renal subcapsular hemorrhage and stable blood pressure control. An abdominal CT (**Figure 3F**) on October 9, 2025 (compared to July 11) demonstrated significant absorption of the hematoma and fluid collection, with no recurrence of renal hemorrhage. Concurrent tumor assessment showed stable lung lesions (**Figure 1F**) and well-controlled brain metastases (**Figure 2C**) after radiotherapy. Based on the treatment course and medication history, this episode of renal subcapsular hemorrhage was clinically considered to be associated with Bevacizumab therapy.

## 3 Discussion

This case involves a patient with EGFR-positive mutant lung adenocarcinoma. After progression on EGFR-TKI therapy, the treatment was switched to a combination regimen containing Bevacizumab. Following the completion of four cycles, the patient was readmitted due to intermittent fever. Investigations ruled out common causes of fever such as infection and revealed concurrent hypertension, anemia, and CT-confirmed subcapsular renal hemorrhage. The fever was considered to be an absorption fever secondary to the renal subcapsular hemorrhage. The patient’s condition improved after discontinuing Bevacizumab and implementing comprehensive management. Therefore, this rare hemorrhagic event was determined to be closely associated with Bevacizumab, with its mechanism likely involving the combined effects of multiple factors. We further analyzed the interrelationship and chronological sequence of the three mechanisms: the patient’s pre-existing bilateral renal cysts represented areas of parenchymal weakness, serving as the baseline predisposing factor; bevacizumab-induced injury to the subcapsular microvascular endothelium and increased vascular fragility acted as the initiating factor; and drug-induced hypertension elevated renal vascular pressure, promoting hemorrhage progression as the contributing factor. So these three factors worked in sequence: first the baseline predisposition from the pre-existing cysts, then the bevacizumab injury as the trigger, and finally the drug-induced hypertension that pushed the bleeding forward. Together, they led to this episode of subcapsular renal hemorrhage.

### 3.1 Direct vascular injury effect of bevacizumab

Vascular Endothelial Growth Factor (VEGF) is a key regulator for maintaining the structural integrity and function of the renal microvasculature, playing a crucial physiological role particularly in glomerular capillary endothelial cells, podocytes, and renal tubular cells (8). By specifically blocking VEGF-A signaling, Bevacizumab directly disrupts the physiological protective mechanisms of the subcapsular micro vessels in the kidney. This leads to insufficient nutritional support for vascular endothelial cells, reducing their reparative capacity and increasing vessel wall fragility, thereby predisposing them to rupture and hemorrhage. Furthermore, VEGF-A blockade activates CD4^+^ and CD8^+^ T cells as well as the complement cascade, exacerbating renal vascular wall damage through immune-mediated inflammatory injury, ultimately increasing the risk of hemorrhage (8–10).

### 3.2 Synergistic role of hypertension

Bevacizumab-induced hypertension is a known vascular-related adverse reaction (5). In this case, after completing four cycles of Bevacizumab-containing combination therapy, sustained elevation in blood pressure was observed (repeated measurements ≥140/90 mmHg, with a peak of 154/87 mmHg), meeting the diagnostic criteria for hypertension. According to literature, persistent hypertension not only damages renal tubular cells, leading to epithelial-mesenchymal transition (EMT) and tubulointerstitial fibrosis but also causes injury at the glomerular level, resulting in changes in post-glomerular capillaries. This process induces endothelial damage and hypoxia (11), thereby increasing the risk of subcapsular renal hemorrhage.

### 3.3 Synergistic effect of underlying conditions

Prior to Bevacizumab treatment, this patient had a pre-existing right renal cyst, and a new left renal cyst was discovered during therapy. The presence of renal cysts suggests possible areas of structural weakness in the renal parenchyma. Under the combined influence of Bevacizumab-induced vascular endothelial dysfunction and drug-related hypertension, such vulnerable areas become more prone to vascular rupture and hemorrhage. Although there is no direct evidence establishing a causal relationship between the underlying renal pathology and the hemorrhage, it may serve as a significant synergistic factor, increasing the patient’s risk of hemorrhage during anti-angiogenic therapy.

Comparison with existing literature highlights the uniqueness of this case. A PubMed search revealed no radiologically confirmed cases of bilateral subcapsular renal hemorrhage directly attributed to bevacizumab. The renal adverse effects of this drug are predominantly proteinuria and hypertension, rather than overt hemorrhage. The uniqueness of this case lies in the absence of common bleeding etiologies such as primary renal tumor, trauma, or coagulopathy, and no recurrence of bilateral subcapsular renal hemorrhage after bevacizumab withdrawal. Nevertheless, this case also has certain limitations. As a single-case report, conclusions should be extrapolated with caution. While common etiologies were excluded as much as possible, interference from other unknown rare factors cannot be completely ruled out. Larger sample studies are needed in the future to further confirm the associated risk factors.

## 4 Conclusion

Based on a clinical case of bilateral subcapsular renal hemorrhage in a lung adenocarcinoma patient during immunotherapy, suspected to be induced by bevacizumab, this report discusses the potential causes and proposes corresponding clinical management recommendations. For cancer patients receiving bevacizumab, routine monitoring of blood pressure, periodic checks of urinalysis, complete blood count, and liver/renal function, combined with dynamic imaging assessment, are essential for early detection of drug-related abnormalities. If patients develop unexplained anemia (e.g., progressive hemoglobin decline), flank/abdominal pain (persistent dull ache or sudden severe pain), or significant blood pressure elevation (reaching hypertensive levels) during treatment, a high suspicion for visceral hemorrhage should be raised. Immediate abdominal CT or ultrasound should be arranged to clarify the bleeding site, extent, and severity. According to bevacizumab prescribing guidelines, patients who experience Grade 3 or 4 hemorrhage during treatment should permanently discontinue the drug. In this case, the subcapsular hemorrhage caused significant clinical symptoms like anemia and required active medical intervention (absolute bed rest, hemostasis, anti-infection) for control, meeting the criteria for an NCI-CTCAE Grade 3 bleeding event. Therefore, strict adherence to the guideline for permanent discontinuation was necessary. A rapid multidisciplinary team (MDT) approach involving urology, radiology, and oncology should be initiated to develop an individualized management strategy. A comprehensive re-assessment of the patient’s condition is crucial to carefully weigh the benefits of subsequent antitumor therapy against the bleeding risk, adjusting the treatment plan if necessary. While bevacizumab plays a vital role in the comprehensive treatment of lung cancer, colorectal cancer, and gynecological cancers, with its survival benefits well-established, its potential for severe hemorrhage cannot be overlooked. Therefore, clinicians must maintain high vigilance, strengthen monitoring and management throughout the treatment course, strive for early recognition, timely drug discontinuation, and standardized management to maximize patient survival benefit while ensuring safety.

## Data Availability

The original contributions presented in the study are included in the article/supplementary material. Further inquiries can be directed to the corresponding author.

## References

[1] Garcia J Hurwitz HI Sandler AB Miles D Coleman RL Deurloo R . Bevacizumab (Avastin®) in cancer treatment: A review of 15 years of clinical experience and future outlook. Cancer Treat Rev. (2020) 86:102017. doi: 10.1016/j.ctrv.2020.102017. PMID: 32335505

[2] Banerjee A Vathiotis I Halder D Sen U Wang X Shields MD . Molecular landscape and therapeutic strategies of lung cancer lineage plasticity. J Thorac Oncol Off Publ Int Assoc For Study Lung Cancer. (2025) 20:1582–93. doi: 10.1016/j.jtho.2025.06.012. PMID: 40751717

[3] Guo SB Hu LS Huang WJ Zhou ZZ Luo HY Tian XP . Comparative investigation of neoadjuvant immunotherapy versus adjuvant immunotherapy in perioperative patients with cancer: A global-scale, cross-sectional, and large-sample informatics study. Int J Surg (London England). (2024) 110:4660–71. doi: 10.1097/js9.0000000000001479. PMID: 38652128 PMC11325894

[4] Succony L Rassl DM Barker AP McCaughan FM Rintoul RC . Adenocarcinoma spectrum lesions of the lung: Detection, pathology and treatment strategies. Cancer Treat Rev. (2021) 99:102237. doi: 10.1016/j.ctrv.2021.102237. PMID: 34182217

[5] Owen JJ Long S Mullinax K Griffin J . Bevacizumab-induced subungual hemorrhage. Dermatol Online J. (2024) 30(5):6. doi: 10.5070/d330564427. PMID: 39680968

[6] Tang L Ding C Li H Yin G Zhang H Liu WS . A pharmacovigilance study of adverse event profiles and haemorrhagic safety of bevacizumab based on the FAERS database. Expert Opin Drug Saf. (2024) 23:213–20. doi: 10.1080/14740338.2023.2248876. PMID: 37581403

[7] Takahashi H Morishita K Okada Y . Renal subcapsular hematoma with abscess in severe COVID-19. IDCases. (2022) 28:e01490. doi: 10.1016/j.idcr.2022.e01490. PMID: 35369569 PMC8958848

[8] Li J Li XL Li CQ . Immunoregulation mechanism of VEGF signaling pathway inhibitors and its efficacy on the kidney. Am J Med Sci. (2023) 366:404–12. doi: 10.1016/j.amjms.2023.09.005. PMID: 37699444

[9] Gu T Jiang A Zhou C Lin A Cheng Q Liu Z . Adverse reactions associated with immune checkpoint inhibitors and bevacizumab: A pharmacovigilance analysis. Int J Cancer. (2023) 152:480–95. doi: 10.1002/ijc.34332. PMID: 36274626

[10] Klomjit N Evans R Le TK Wells SL Ortega J Green-Lingren O . Frequency and characteristics of chemotherapy-associated thrombotic microangiopathy: Analysis from a large pharmacovigilance database. Am J Hematol. (2023) 98:E369–e72. doi: 10.1681/asn.20233411s1782c PMC10844958 37740927

[11] Costantino VV Gil Lorenzo AF Bocanegra V Vallés PG . Molecular mechanisms of hypertensive nephropathy: Renoprotective effect of losartan through Hsp70. Cells. (2021) 10:3146. doi: 10.3390/cells10113146. PMID: 34831368 PMC8619557

